# Biochemical Traits, Survival and Biological Properties of the Probiotic *Lactobacillus plantarum* Grown in the Presence of Prebiotic Inulin and Pectin as Energy Source

**DOI:** 10.3390/ph5050481

**Published:** 2012-05-15

**Authors:** Filomena Nazzaro, Florinda Fratianni, Pierangelo Orlando, Raffaele Coppola

**Affiliations:** 1 Istituto di Scienze dell’Alimentazione, ISA-CNR,Via Roma 64, 83100, Avellino, Italy; Email: fratianni@isa.cnr.it (F.F.); direttore@isa.cnr.it (R.C.); 2 Istituto di Biochimica delle Proteine, IBP-CNR, Via P. Castellino 121, 80124, Napoli, Italy; Email: p.orlando@ibp.cnr.it (P.O.)

**Keywords:** probiotics, prebiotics, microelectrophoresis, antioxidative, aggregation, short chain fatty acids

## Abstract

The viability of the probiotic strain *Lactobacillus plantarum subsp. plantarum*, after its passage through simulated gastric and pancreatic juices, was evaluated as function of its pre-growth in a medium containing the known prebiotics pectin or inulin, and was compared to glucose used as control. The presence of pectin or inulin did not markedly affect the growth (10.07 log_10_ colony forming units/mL and 10.28 log_10_ colony forming units/mL for pectin and inulin respectively *versus* 10.42 log_10_ colony forming units/mL obtained for glucose). Pectin and inulin, in contrast to glucose, induced cell stress resistance against gastrointestinal juices (Δ log_10_1.5 and 2.4 colony forming units/mL respectively, *versus* Δ log_10_ 4.0 for glucose). The data were corroborated by the analysis of the protein pattern following stress treatments which, in the case of microbial cells grown with glucose, revealed a more marked protein degradation after the double passage through simulated gastric and intestinal juices. Inulin stimulated the production of the relevant healthy bio-molecule butyrate, which amount was 30% higher respect of growth in the presence of glucose. Inulin and pectin improved cell DPPH scavenging activity, and an impressive hydrophobicity (35.28% and 34.81%, respectively) was observed with respect to the microbial growth in presence of glucose (3.39%).

## 1. Introduction

The human gastrointestinal microflora, at present referred to as “microbiota” is a complex ecosystem of approximately 300–500 bacterial species comprising nearly two million genes (the so called “microbiome”). Gut microbiota is one of the most important key factors in maintaining and preserving the health of the entire body. Consequently, its modification can negatively influence the well-being of the host in different modes. To prevent such events, the intestinal ecosystem needs to be retained in a “healthy” state through a constant supply of functional ingredients, such as probiotics and prebiotics. Probiotics, derived from the Greek and meaning “for life”, is a term used to define living non-pathogenic organisms which have a demonstrated beneficial effect on the host when ingested in adequate amounts [1]. The most commonly used probiotics are represented by lactobacilli, bifidobacteria and non-pathogenic yeasts. They must be capable of surviving the passage through the stomach to enter the intestinal tract, where they exert helpful effects on the gut microbiota, and of resisting to the stressful environments during industrial processes. The most important and known beneficial effects of probiotics include the prevention of diarrhea, constipation, and food allergies; other factors, such as a reduction of gas production, changes in bile salt conjugation, anti-bacterial, anti-inflammatory and anti-viral effects, may result relevant to the benefits of specific probiotics [2]; on the other hand, they contribute to the synthesis of nutrients and to enhancement of their bioavailability, and have anti-carcinogenic properties [3]. Some probiotics have also a documented high total antioxidative activity (TAA) and total antioxidative status (TAS) of their intact cells and lysates, and are characterized by a complete glutathione system; thus, they can help in limiting the negative effects of the so called Reactive Oxygen Species (ROS), whose high levels can cause damage to DNA, lipids, proteins or carbohydrates, and contribute to age-related disorders, such as cancer, atherosclerosis and hypertension [4,5,6]. Adhesion to the intestinal epithelial cells is another well known important prerequisite for colonization of probiotic strains in the gastrointestinal tract, that contributes to prevent their immediate elimination by peristalsis and provide a competitive advantage to the gut microbiota. To provide the desired benefits, probiotics should persist in the product as viable cells during the entire shelf life. Different reports suggest that the minimum necessary dose suitable to ensure a therapeutic effect should ranges between 8 and 9 log colony forming units (cfu)/mL. Prebiotics are non-digestible food ingredients that beneficially affect the host’s health by selectively stimulating the growth and/or activity of some genera of microorganisms in the colon, generally lactobacilli and bifidobacteria [7]. Due to their chemical structure, prebiotics are not absorbed in the small intestine, but are fermented and used in the colon by endogenous bacteria as a source of energy, and metabolic substrates, including lactic and short-chain fatty acids (SCFAs) and miscellaneous gases, with diverse biological roles, are the end products of their fermentation. The occurrence of prebiotics may inhibit pathogen adhesion [8], modulate lipid metabolism [9], decrease the possibility of colon carcinogenesis, enhance mineral absorption [10], and control the secretion of gastrointestinal peptides which are implicated in appetite regulation [11]. Several prebiotics are effective agents in dental caries prevention and in the facilitation of the mineral absorption; they can also act as antioxidants, antibiotic alternatives, regulators of blood glucose in diabetics and serum lipids in hyperlipidemics [12]. Inulin and pectin are two of the most used prebiotics. They resist digestion by gastric and pancreatic enzymes both *in vitro* and *in vivo* [13]. Inulin is a natural polymer formed by fructose units that are joined by a β (2→1) glycosidic bond. It is one of the best known prebiotic oligosaccharides with recognized specific and different functional attributes, such as modulation of the gut microbiota, prevention of adhesion and colonization by pathogens, stimulation of anti-inflammatory effects, reduction of food intake, modulation of bowel movements, regulation of alterations in lipid and glucose metabolism. Pectins, complex polysaccharides containing 1,4-linked α-D-galacturonic acid residues, are highly fermentable substances responsible for increases in the fecal bulk [14], and exhibit bifidogenic and generally prebiotic properties on different strains of probiotic microorganisms. The aim of the present work was to evaluate the effectiveness of inulin and pectin to preserve the viability and the protein pattern of the probiotic strain *Lactobacillus plantarum* subsp. *plantarum* through the gastrointestinal tract, when the strain was pre-grown with these two prebiotics as carbon source, compared to glucose used as control. In order to gain information on the influence of prebiotics on the *its* potential structural properties, that are responsible for adhesion, and on some aspects of the microbial metabolic pathway, the *in vitro* hydrophobicity and the production of some short chain fatty acids by *L. plantarum subsp. plantarum* were evaluated, respectively. Finally, the potential radical scavenging capability of the strain was assessed.

## 2. Materials and Methods

### 2.1. Strain and Culture Conditions

*L. plantarum* subsp. *plantarum* DSM 20174 is the DSMZ reference strain (Deutsche Sammlung von Mikroorganismen und Zellkulturen GmbH, Braunschweig, Germany). Cells were anaerobically grown at 30 °C for 24 h in MRS deMan, Rogosa and Sharpe broth [15] (casein peptone, tryptic digest 10 g/L; meat extract 10 g/L; yeast extract 5 g/L; Tween 80 1 g/L; K_2_HPO_4_ 2 g/L; Na-acetate 5 g/L; (NH_4_)_2_ citrate 2 g/L; MgSO_4_ × 7H_2_O 0.20 g/L; MnSO_4_ × H_2_O 0.05 g /L), pH 6.2, complemented with 20 g/L of inulin, pectin or glucose, the last used as control. All chemicals were supplied by Sigma (Milano, Italy). All solutions were sterilized and all manipulations were aseptically performed.

### 2.2. Resistance to Simulated Gastric and Intestinal Juices

The artificial gastric juice contained MRS broth, plus 3 g/L pepsin, with a final pH adjusted to 2.0. The artificial intestinal juice contained MRS broth plus 1 g/L pancreatin and 4.5 g/L bile salts (sodium glycocholate and sodium taurocholate, code number X606C, Oxoid, Basingstoke, UK). Both solutions were filter-sterilized (0.22 μm, Millipore SpA, Milano, Italy). The strain was treated using the protocol of De Giulio *et al*. [16]. Bacterial suspensions were incubated in artificial gastric juice for 180 min at 30 °C and recovered by centrifugation. After washing with sterile physiological solution (8.5 g/L NaCl), pellet was resuspended in the simulated gastrointestinal juice (4.5 mL/100 μL of cells). Samples were incubated at 30 °C for 60 min. Cell viability (cfu/mL) was evaluated by anaerobic culturing on MRS plates (30 °C, 48 h) before and after incubation in the two simulated gastrointestinal juices. Each separate experiment was performed three times.

### 2.3. Preparation of Heat Killed Cells

Preparation of Heat Killed Cells (HKC) was performed following the method described by Liu *et al*. [17] with some modifications. Two percent of *L. plantarum* *subsp. plantarum* was inoculated into MRS broth (Sigma Aldrich Italia, Milano, Italy) and cultured at 30 °C for 20 hr under anaerobic conditions. Cells were harvested by centrifugation for 5 min at 5,000*× g* at 4 °C (Biofuge, Beckmann Coulter Italia,Cassina de Pecchi, Italy); the pellet was washed twice, resuspended in phosphate-buffered saline (pH 7.2) buffer, heated at 100 °C for 30 min and dried. Samples were re-suspended again to 5 mg dry cell/mL, autoclaved at 121 °C for 15 min and stored in 4 °C prior to use.

### 2.4. Biochemical Analysis

#### 2.4.1. Protein Profile

Cells were centrifuged (11,600 × g, 4 °C, 15 min; Heraeus, Cavenago Brianza, MI, Italy) and washed twice in 0.05 M Tris–HCl, pH 7.4 (1:5 w:vol). Pellets were re-suspended in 300 μL 0.05 M Tris–HCl pH 7.4 with 2% SDS. Following the addition of glass beads, samples were continuously mixed for 5 min, then boiled at 100 °C for 3 min [18]. Glass beads and cell debris were removed by centrifugation, and the supernatant was collected. The protein content of the supernatant was evaluated according to Bradford [19]. A 5-μL aliquot of each sample was diluted with 84 μL of ultrapure water and 2 μL of sample buffer (Experion^TM^ Pro260 kit, Bio-Rad, Hercules, CA, USA) containing 1 μL of β- mercaptoethanol. All samples were treated at 100 °C for 3 min. A 6-μL sample was analyzed by chip capillary electrophoresis (Experion^TM^ Pro260 Analysis Kit) over a range of molecular weights from 1.2 and 260 kDa. The analysis was completed using an Experion^TM^ automated electrophoresis system (Bio-Rad Hercules, CA, USA) and the appropriate software (fluorescence detection with a 10-mW semiconductor laser emitting at 630 nm). The automated analysis took 30 min for a set of 10 samples. 

#### 2.4.2. Production of Short Chain Fatty Acids

The production of acetic, lactic and butyric acid was evaluated in the filtered supernatant obtained from microbial culture following growth at 30 °C for 24h in the presence of pectin, inulin or glucose, before the microbial incubation in simulated gastrointestinal juices. The analysis was performed by high-performance liquid chromatography [18,20] using a Gold System apparatus with an ultraviolet detector (Beckman Coulter Italia, Cassina de Pecchi, Italy). Samples were loaded onto a pre-packed Aminex HPX-87 column (300 × 7.8 mm, Bio-Rad) and eluted with 0.005 M sulphuric acid. The total running time was 40 min, the flow rate was 0.6 mL/min, the injection volume was 20 μL, and the detection wavelength was set at λ=210 nm. 

#### 2.4.3. Free Radical–Scavenging Capacity

The free radical–scavenging activity of HKC was measured with the stable radical 2,2-diphenyl-1-picrylhydrazyl (DPPH)[21]. The analysis was performed by adding 50 and 100 μl of HKC to a DPPH methanol solution (153 mM). Then, the absorbance was spectrophotometrically measured (Cary 50 Uv/Vis ,Varian-Agilent Italia, Cernusco sul Naviglio, Italy) at λ= 517 nm. The absorbance of DPPH without antioxidant (control sample) was used as a baseline measurement. The capability of the test material to scavenge DPPH radicals was calculated as percentage compared to a blank, containing methanol, after a 60-minute incubation, by the formula:

DPPH-scavenging activity (%) = [A_517_ (sample T0)- A _517_ (sample T 60’) / A _517_ (sample T0)] × 100

#### 2.4.4. Microbial Adhesion to Solvent

The microbial adhesion to hydrocarbons (MATH) test was performed according to Rosenberg *et al*. [22] with some modifications. Cells of *L. plantarum* *subsp. plantarum* were washed with sterile phosphate buffered solution (pH 7.2), harvested and re-suspended in the same buffer. The absorbance of the cell suspension was measured at 600 nm (A0), then an equal volume of xylene was added. The two-phase system was thoroughly mixed by vortexing for 3 min. The aqueous phase was accurately removed after 1 h of incubation at 37 °C and its absorbance (A1) was measured. The adhesion was calculated from three replicates as percentage decrease in the optical density of the original bacterial suspension by the formula:

[A0-A1/A0] × 100.

### 2.5. Statistical Analysis

Three replicates were carried out for each analysis. Statistical significance was performed as described in De Giulio *et al*. [16] and set at P < 0.05.

## 3. Results and Discussion

### 3.1. Culture Growth and Resistance to Gastrointestinal Stress

Several species of the gastrointestinal microbioma have the capability to ferment oligo- and polysaccharides [23]. Such capacity is common within the genus *Bifidobacterium*, but it is strain-dependent among the genus *Lactobacillus* [24]. 

**Figure 1:**
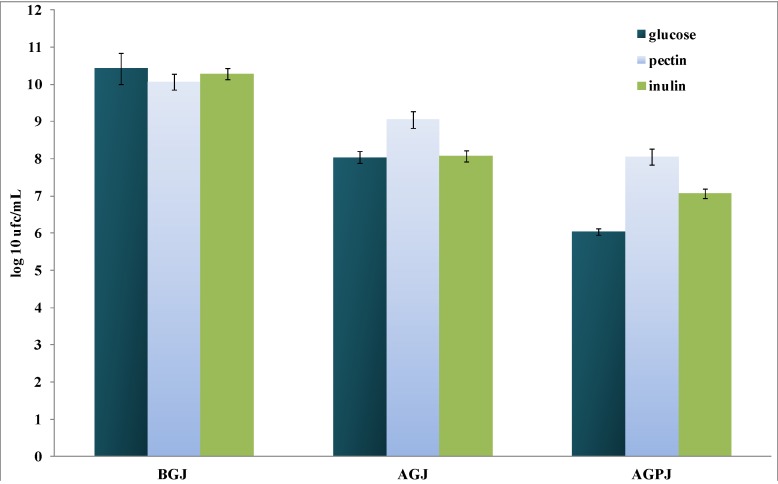
The viability of *L. plantarum* *subsp. plantarum* grown in the presence of 20 g/L of glucose, pectin, or inulin, before (BGJ) and after passage in simulated gastric (AGJ) followed by pancreatic (AGPJ) juices. The data are expressed as log_10_ cfu/mL (mean ± SD, n = 3). Statistical significance was set at P <0.05. For the details, see materials and methods.

In our experiments, *L. plantarum* *subsp. plantarum* was capable of growing on pectin and inulin, reaching values of 10.07_log10_ cfu/mL (±0.03) and 10.28_log10_ cfu/mL (±0.06), respectively (Figure 1). These values were not consistently different with respect to that obtained in glucose (used as control), 10.42_log10_ cfu/mL (±0.05), although lower with respect to those observed when *L. acidophilus* was treated undeer the same growth conditions [25]. In contrast, the tolerance to the simulated gastric and pancreatic juices was deeply dependent on the initial growth conditions, which caused a more effective viability when the strain was pre-treated in the presence of pectin or inulin (Δ log_10_ 1.5 and 2.4 cfu/mL respectively at P < 0.05) as compared to the growth in presence of glucose (Δlog_10 _4.0 cfu/mL at P < 0.01) (Figure 1). The evident loss of viability confirms once more that glucose, different from inulin and pectin, would be not capable to exercise a significant defensive effect, mainly after the double passage through the simulated gastric and pancreatic juices. 

**Figure 2:**
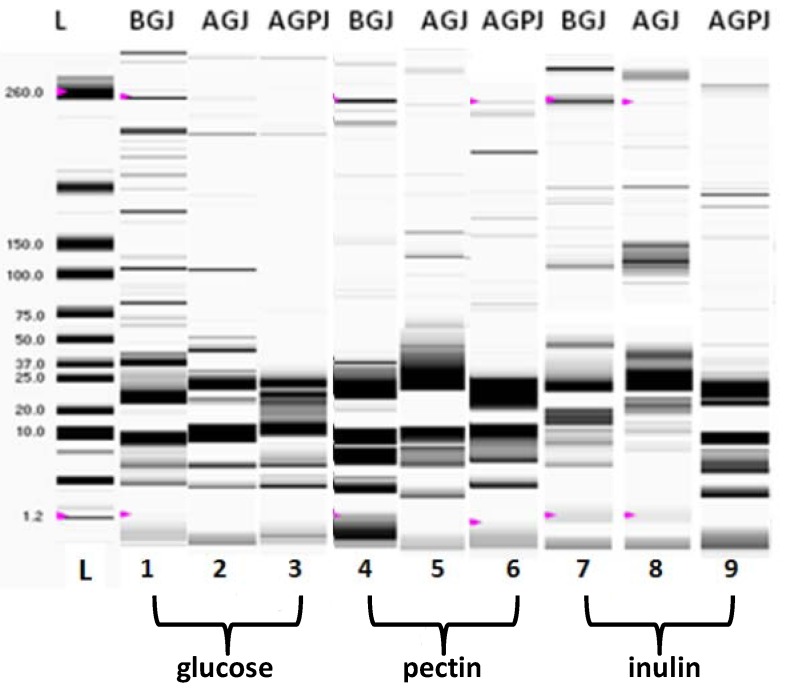
The protein profile of *L. plantarum* *subsp. plantarum* grown in the presence of 20 g/L of glucose (lanes 1-3), pectin (lanes 4-6), or inulin (lanes 7-9), before (BGJ) and after passage in simulated gastric (AGJ) followed by pancreatic (AGPJ) juices. Profiles, shown as gel like data, were obtained through microelectrophoresis on chip. L indicates the MW standards (kDa). For the details, see materials and methods.

The diverse resistance in the simulated gastro-intestinal juices could be also linked to the difference on the protein profiles, whch were analyzed by the lab-on-a-chip approach (Figure 2). 

Growth in the presence of pectin (Figure 2, lanes 4-6) or inulin (Figure 2, lanes 7-9) beneficially influenced the preservation of protein expression patterns, mainly during the subsequent passages of the strain through the simulates of gastric (AGJ) and pancreatic (AGPJ) juices, if compared to the growth in presence of glucose used as control (Figure 2, lanes 1-3): in this last case, it gave rise to a disappearance of bands greater than 37 kDa, although the effect was less marked respect to *L. acidophilus* [25]. While we don’t know if and in which manner such proteins could be involved in the resistance of microorganism to those stress conditions, the degradation of the protein profile might be ascribable to the increase in the mortality of the cell population and can qualitatively be related to the data obtained by the enumeration of the colony forming units.

### 3.2. Production of Short-Chain Fatty Acids (SCFAs)

Fermentation of carbohydrates in the colon by the intestinal microbiota and probiotics leads to production of short-chain fatty acids (SCFA) such as acetate and butyrate and gases (mainly CO_2_ and H_2_) [26]. SCFAs are quickly used by gut microbiota or absorbed (through the mucosal surface of the caecum and ascending colon) and utilized as energy sources by the host. Mucosal cells efficiently use butyrate to preserve and maintain their structure. Acetate goes across the intestinal wall without being metabolized much and is carried to the systemic circulatory system, reaching the blood and entering the liver [27]. The capacity of certain intestinal microbes to utilize carbohydrates not digested by the host plays a key part in their effectiveness to adequately colonize the mammalian gastrointestinal tract. The adaptation of *L. plantarum subsp. plantarum* to a variety of environmental niches can be associated with its diverse catabolic potential, demonstrating the competitive benefits of utilizing complex sugars [28]. The amount of lactate, acetate and butyrate produced by *L. plantarum subsp. plantarum* grown in the presence of glucose, pectin or inulin, was evaluated by HPLC and is shown in Table 1.

**Table 1:** *L. plantarum* *subsp. plantarum* production of lactic, acetic and butyric acid during growth in the presence of 20 g/L of glucose, pectin or inulin The data are expressed as μmoles/mL (n = 3), ND: not detectable. For the details, see materials and methods.

	Acetic acidμmol/mL ± SD	Lactic acidμmol/mL ± SD	Butyric acidμmol/mL ± SD
Glucose	276 ± 12	213 ± 25	7.5 ± 0.52
Inulin	208 ± 20	162 ± 20	10.5 ± 0.65
Pectin	213 ± 70	147 ± 10	ND

The presence of glucose gave rise to higher amounts of acetate respect to inulin. Probably, the presence of inulin promoted the production of butyrate, which amount was higher (about 30%) respect to glucose, used as control. Conversely to *L. acidophilus* grown in presence of the same substrates [25],pectin, although it stimulated the production of acetate and lactate, did not induce the strain to produce detectable levels of butyrate, confirming the hypothesis that different carbohydrates might affect the metabolic pathways in lactobacilli in diverse ways. In any case, the production of butyrate may be considered a result of biological relevance, based on its known healthy effects: in fact, it is the preferred energy source for colonic epithelial cells, and has anti-carcinogenic and anti-inflammatory effects [29]; butyrate has been found to slow proliferation and promote the expression of phenotypic markers of differentiation in different cancer cell lines and *in vivo* [30,31,32]. Our data suggest that the presence of inulin in the growth medium of *L. plantarum* *subsp. plantarum* might induce an up-regulation of butyrate production; however, conversely to *L. acidophilus* grown under the same conditions [25], such a capability cannot be correlated to the higher resistance exhibited by the strain through its passage the gastrointestinal juice, as demonstrated by the absence of butyrate production after growth of *L. plantarum subsp. plantarum* in the presence of pectin. 

### 3.3. Antioxidative Activity

Reactive oxygen species (ROS), are formed in the process of incomplete reduction of oxygen. Physiological concentrations of ROS are needed for a regular functioning of the cell and for the production of energy; indeed, they are involved in phagocytosis and in the regulation of the mechanisms involved in intercellular signaling [33]; however, high levels of ROS may lead to damage of the host’s DNA, proteins, carbohydrates and lipids [34]. Different studies on the antioxidant activity of lactic acid bacteria have demonstrated that some strains are capable of decreasing the risk of ROS accumulation and degrading superoxide anion and hydrogen peroxide. Kaizu *et al*. first reported the antioxidant activity of *Lactobacillus* sp SBT 2028[35]; Lin and Yen indicated the intracellular cell-free extracts of *B. longum* ATCC 15708 and *L. acidophilus* ATCC 4356 have antioxidant activity [36]; other LAB strains, such as *L. fermentum* E3, *L. fermentum* E18 and *L. acidophilus* ATCC 4356, could overcome the oxidative stress [6,37,38]. In our study, antioxidant activity was exhibited by the HKC of the *L. plantarum subsp. plantarum* grown with the three carbohydrates as energy source. The activity (16.45% ± 1.06) was generally in the range observed for other *Lactobacillus* strains [39]. The presence of inulin and pectin increased in an impressive manner such activity compared to glucose, and ten times higher (24.5 % ± 1.04, and 31.5% ± 2.75, respectively) antioxidant activity values were observed. This aspect can be of particular relevance, taking into account that probiotic strains with high antioxidative activity might not only contribute to fighting all diseases related to ROS, but also they can exhibit, *in vivo*, a greater survival in a particular ecological niche and, probably also through the production of organic acids, a high rank of anti-infectious potential, as demonstrated by Hutt *et al*. [39] and Truusalu *et al*. [40] in different probiotics. 

### 3.4. Microbial Adhesion to Solvent

Cell adhesion is a complex process implicating contact between the bacterial cell membrane and the interacting surfaces. The capacity of adhesion to epithelial cells and mucosal surfaces has been suggested to be an important property of many bacterial strains used as probiotics. Several studies have investigated the composition, structure and forces involved in bacterial adhesion to intestinal epithelial cells [41] and mucus [42]. The microbial adhesion to hydrocarbons (MATH) test has been extensively used for measuring cell surface hydrophobicity/hydrophilicity in lactic acid bacteria [43]. The test was used to evaluate the hydrophobic/hydrophilic cell surface properties of *L. plantarum subsp. plantarum,* deriving from the presence of inulin, pectin or glucose used as source of energy. After growth in the presence of inulin and pectin, microbial cells adhered to xylene, with percentages of 34.81 % (± 3.14) and 35.28% (± 2.75), respectively, resulting 10 times higher than that observed after growth with glucose (3.39 % ± 0.012). In probiotics, a good correlation between surface hydrophobicity and the capacity to adhere to the intestinal mucosa has been ascertained [44,45]: in fact, these bacteria, also through such a structural property, are capable of interacting more effectively with the intestinal mucosa. The fact that a much higher percentage of *L. plantarum subs plantarum* cells adhered to the apolar solvent xylene after growth in presence of the two prebiotics, compared to the microbial growth with glucose, demonstrated that these two prebiotics could positively affect the hydrophobicity of the cell surface of this strain; thus, pre-incubation of the strain in the presence of the two prebiotics might render its cell surface potentially more capable of colonizing the intestine. Indeed, our results confirm that hydrophobicity index might be dependent on the carbon source used as energy substrate [46].

## 4. Concluding Remarks

The results shown in this study demonstrate the capability of *Lactobacillus plantarumsubsp. plantarum* to ferment inulin and pectin; the two prebiotics could be considered good candidates to improve the microbial resistance to the adverse conditions present in the body, enhancing its survival through the gastrointestinal tract; indeed, they can ameliorate its capability to adhere to the intestinal mucosa. The behavior exhibited by the strain grown in the presence of the two prebiotics could represent a valid support in contrasting the negative effects of high amount of ROS; indeed, use of the prebiotic inulin might stimulate the strain to produce higher amount of some bio-components, such as butyrate, with beneficial effects for human health. The identification of the best growth conditions for screening *Lactobacillus* strains according to their activity under various environmental conditions could precede the clinical efficacy studies for adjunct treatment with probiotics in cure of different gastrointestinal and urinary tract infections as well as in a more efficient support by these microorganisms to human health and well being.
